# Prognostic value of the systemic inflammation response index in patients with acute ischemic stroke

**DOI:** 10.1002/brb3.2619

**Published:** 2022-05-19

**Authors:** Yaping Zhou, Yidi Zhang, Mingming Cui, Yuming Zhang, Xiuli Shang

**Affiliations:** ^1^ Department of Neurology The First Hospital of China Medical University Shenyang China; ^2^ Department of Rehabilitation Medicine Affiliated Tenth People's Hospital of Tongji University Shanghai Tenth People's Hospital Shanghai China

**Keywords:** acute ischemic stroke, inflammation, prognosis, systemic inflammation response index

## Abstract

**Objectives:**

Inflammation plays an essential role in acute ischemic stroke (AIS). Recent studies have recognized the systemic inflammation response index (SIRI) as a useful index to indicate inflammation status and predict the prognosis of multiple diseases. However, the relationship between SIRI and AIS prognosis is unclear. Our study is aimed to investigate the association between SIRI and the prognosis of AIS.

**Methods:**

Our study prospectively recruited 287 consecutive patients with first‐ever stroke within 72 h after stroke. Demographic and clinical information was collected at baseline. The functional prognosis was assessed 3 months after AIS using the modified Rankin Scale (mRS). A poor outcome was defined as mRS > 2. SIRI was calculated as neutrophil × monocyte/lymphocyte count. Univariate and multivariate analyses were introduced to identify the association between SIRI and AIS prognosis. Receiver operating characteristic curve and reclassification analyses were used to evaluate the predictive value of SIRI for AIS prognosis.

**Results:**

The patients with poor prognosis account for 27.5% of all participants. After fully adjusting for all covariates, each standard deviation increment of SIRI caused 58.9% additional risk for poor prognosis after AIS. When dividing SIRI into quartiles, the fourth quartile had a 6.152 times risk than the first quartile. Moreover, after adding SIRI into established clinical risk factors, AUC showed a significant improvement (0.829 vs. 0.790, *p* for comparison = .016). Consistently, category‐free net reclassification index (NRI, 0.761, 95% CI: 0.517–1.004, *p* < .001) and integrated discrimination index (IDI, 0.093, 95% CI: 0.0512–0.134, *p* < .001) confirmed the improvement by SIRI to predict poor prognosis of AIS,

**Conclusion:**

SIRI is an independent prognostic indicator for AIS. Elevated SIRI is associated with poor functional outcome of AIS. Our findings suggest the usefulness of SIRI to refine the risk stratification of unfavorable prognosis of AIS.

## Graphical abstract

The present study investigates the association between SIRI and the prognosis of AIS. Our results demonstrated that elevated SIRI is independently associated with poor functional outcome of AIS, which implicates the usefulness of SIRI to refine the risk stratification of unfavorable prognosis of AIS.

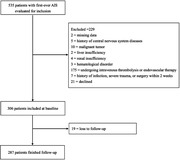

## 1 INTRODUCTION

With an increased incidence and prevalence, stroke has become the primary cause of mortality and the most critical disease resulting in reduced disability‐adjusted life years in China (Wu et al., 2019). Ischemic stroke accounts for more than 70% of all strokes and is a major cause of disability, causing a significant burden on human health and the economic (Campbell et al., 2019). Therefore, finding useful biomarkers to improve the risk stratification and prevent poor prognosis of acute ischemic stroke (AIS) is needed.

Inflammation has been recognized as a crucial pathological process of cerebral ischemic injury (de Los Rios la Rosa et al., 2012; Jenny et al., 2019; Kim et al., 2016). Inflammatory factors play an indispensable role in the occurrence and development of AIS (Esenwa & Elkind, 2016). In the experimental model of AIS and clinical stroke patients, immune‐inflammatory cells, including neutrophil, lymphocyte, and monocyte, have been found to infiltrate ischemic brain tissue (Gelderblom et al., 2009; Kim et al., 2016; Zrzavy et al., 2018). These inflammatory cells play different roles during AIS. Peripheral circulating neutrophils could enter the brain parenchyma within 6 h after ischemia onset, aggravating cell necrosis and apoptosis in the ischemic area directly by releasing oxygen free radicals (Herz et al., 2015; Matsuo et al., 1995; Zhang et al., 1994). Besides, neutrophil has proved to be a critical source of matrix metalloproteinase‐9 (MMP‐9) within 24 h of AIS, while MMP‐9 is closely related to blood–brain barrier destruction, brain tissue edema, hemorrhagic transformation, and neurological impairment after AIS (Castellanos et al., 2003; Montaner et al., 2001). Monocyte is another essential trigger of inflammation after AIS. After the onset of AIS, peripheral monocytes increase and migrate to the ischemic cerebral tissue, expanding the injured area (Kaito et al., 2013). Unlike neutrophils and monocytes, some specific lymphocytes, such as T‐reg cells, are the primary brain‐protective immunomodulatory cells after AIS (Liesz et al., 2009). Despite experimental evidence for an intimate relationship between neutrophil, monocyte, lymphocyte, and AIS, clinical studies about the relationship between these cells and AIS outcomes are inconsistent. The combined effect of these inflammatory cells on the prognosis of AIS remains unclear.

Recently, a novel indicator of systemic inflammation, systemic inflammatory response index (SIRI), has been proposed as an integration of neutrophil, monocyte, and lymphocyte (SIRI = neutrophil count × monocyte count/lymphocyte count) (Qi et al., 2016). SIRI has been proved to effectively reflect the inflammatory status and predict the prognosis of multiple diseases, including aneurismal subarachnoid hemorrhage and several cancers (Chen et al., 2019; Geng et al., 2018; Li et al., 2017; Pacheco‐Barcia et al., 2020; Qi et al., 2016; Xie et al., 2018; Zhang et al., 2020; Zheng et al., 2019). However, the potential relationship between SIRI and the outcome of AIS is unclear. As we mentioned, experimental research indicated that neutrophil and monocyte could aggravate the damage of AIS. At the same time, lymphocyte showed a neuroprotective effect. Consequently, the increment of SIRI may suggest a more substantial damaging effect and a relatively weaker protective effect (Kaito et al., 2013; Liesz et al., 2009; Matsuo et al., 1995). Therefore, there may be some correlations between SIRI and AIS. The present study is aimed to evaluate the association between SIRI and the prognosis of AIS and explore the prognostic predictive value of SIRI for poor prognosis after AIS.

## 2 MATERIALS AND METHODS

### 2.1 Study population

We prospectively collected the demographic and clinical data of AIS patients admitted to the Neurology Department of the First Hospital of China Medical University from March to August 2020. Medical history information was obtained orally from the patients or their relatives or friends. AIS was defined according to the World Health Organization Multinational Monitoring of Trends and Determinants in Cardiovascular Disease (WHO‐MONICA) criteria and was confirmed by magnetic resonance imaging (MRI) or computed tomography (CT) within 24 h after admission. Inclusion criteria are as follows: (1) age ≥18 years; (2) first ever stroke; (3) admitted within 72 h of initial symptom onset, confirmed by MRI or CT. Exclusion criteria included: (1) absence of data; (2) defined history of infection, severe trauma, or surgery within 2 weeks before AIS onset; (3) patients with central nervous system diseases, cancer, severe liver, or kidney diseases; (4) hematological disorder; (5) patients undergoing intravenous thrombolysis or other endovascular therapy; (6) lost to follow‐up.

This study was prospective research. Written informed consents were obtained from all patients. This study was approved by the Ethics Committee of the First Hospital of China Medical University (No. 2020‐37‐2) and adhered to the tenets of the Declaration of Helsinki.

### 2.2 Clinical data

Baseline clinical data were collected for all patients within 24 h of admission. Blood samples were obtained in the morning after fasting for 8 h. Standard laboratory methods determined the serum levels of neutrophils counts, monocyte counts, lymphocyte counts, fasting plasma glucose (FPG), triglyceride (TG), total cholesterol (TC), low‐density lipoprotein cholesterol (LDL‐C), high‐density lipoprotein cholesterol (HDL‐C), and homocysteine (Hcy) levels. We also recorded the demographic information, including age, gender, race, and risk factors, including body mass index (BMI), hypertension, diabetes mellitus (DM), atrial fibrillation (AF), coronary vascular disease (CVD), peripheral artery occlusive disease (PAO), smoking, and alcohol drinking (Kim et al., 2012). Stroke severity was evaluated using the National Institutes of Health Stroke Scale (NIHSS) (Brott et al., 1989). AIS subtypes were classified into large artery atherothrombosis (LA), cardioembolism (CE), small‐artery occlusion (SA), and others according to the Trial of Org 10,172 in acute stroke treatment (TOAST) (Adams et al., 1993).

### 2.3 Definition

The race was classified into the Han nationality and other nationalities. BMI was calculated as weight (kg)/height (m^2^). Hypertension was determined if systolic blood pressure (SBP) ≥ 140 mmHg and/or diastolic blood pressure (DBP) ≥90 mmHg or current use of antihypertensive medication (Chobanian et al., 2003). Diabetes was defined as FPG ≥7.0 mmol/L or current use of antidiabetes medicines (American Diabetes Association, 2019). Smoking refers to having at least 100 cigarettes in life (Zhou & Shang, 2020). Drinking refers to having at least 12 drinks of any type of alcoholic beverage in the past year (Zhou & Shang, 2020). SIRI was defined as SIRI = neutrophil count × monocyte count/lymphocyte count (Qi et al., 2016).

### 2.4 Prognostic outcomes

All patients were followed up via telephone 3 months after stroke. The functional outcome was evaluated with the modified Rankin Scale (mRS, scores range from 0 to 6). The neurologist responsible for following up was blind to the baseline data of the patients. A poor outcome was defined as mRS > 2 points.

### 2.5 Statistical analysis

Statistical analyses were performed using SPSS for Windows (version 25.0, IBM Corp., Armonk, NY, USA), statistical software packages R (http://www.R‐project.org, The R Foundation), and MedCalc 12.5 (MedCalc Software, Ostend, Belgium). According to the mRS score, all patients were divided into a good outcome group (mRS ≤ 2) and a poor outcome group (mRS > 2). Continuous variables were expressed as mean with standard deviation (SD, normal distribution) or median with interquartile range (non‐normal distribution). Categorical variables were displayed as percentages and a 95% confidence interval (CI). Student's t‐test (or Mann‐Whitney test) and the chi‐square (χ^2^) test (or Fisher's exact tests) were conducted to compare continuous and categorical variables between two groups, respectively. ANOVA test was employed for comparison between multiple groups.

Univariate and multivariate logistic regression were used to analyze the correlation between SIRI and AIS prognosis. The covariables in the multivariate logistic regression model were chosen according to three criteria: first, variables that have been identified as risk factors for stroke; second, variables that were recognized as factors associated with stroke in previous studies; third, variables that showed significant association with SIR or poor outcome of AIS in univariate regression analyses. The results were expressed as odds ratio (OR) and 95% CI.

Receiver operating characteristic curve (ROC) analysis, category‐free net reclassification improvement (NRI), and integrated discrimination improvement (IDI) were performed to investigate the potential value of SIRI to predict poor outcome of AIS (mRS > 2). A two‐tailed *p* value < .05 was regarded as statistical significance.

## 3 RESULTS

**FIGURE 1:**
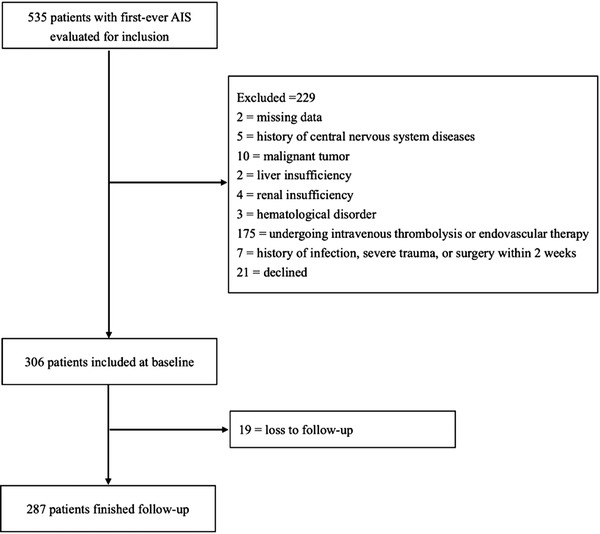
Study profile/flow chart of the study

From March to August 2020, 535 consecutive patients with a first onset AIS were screened, and 306 patients were included in this study at baseline. Nineteen patients were lost to follow up. Finally, 287 patients were eligible for analyses (Figure 1). The characteristics of the study population are summarized in Table 1. The group with poor outcomes had 27.5% of the participants (mRS > 2). Compared with the good outcome group, the poor outcome group had a higher age level, higher NIHSS score, and higher CVD and AF prevalence (all *p* values < .05). As for the laboratory data, the poor outcome patients possessed a higher level of neutrophil counts, lymphocyte counts, fasting blood glucose count, and serum Hcy compared with the good prognosis group (all *p* values < .05). According to the TOAST standard, LA accounted for 61.3% of all patients, CE accounted for 2.8%, SA accounted for 35.2%, and other determined or undetermined etiology accounted for 1.7%. The difference in prognosis among different stroke subtypes was significant (*p* < .001). Finally, the SIRI level in the poor prognosis group was significantly higher than that in the good prognosis group (*p* < .001).

**TABLE 1:** Baseline characteristics of subjects divided by mRS

Variables	All, *n* = 287	mRS ≤2 (*n* = 208)	mRS > 2 (*n* = 79)	*p*
Age (year)	61.55 ± 13.08	59.37 ± 13.08	67.29 ± 11.27	<.001
Gender (male, *n*, %)	198 (69.0%)	149 (71.6%)	49 (62.0%)	.116
Race (Han, *n*, %)	267 (93.0%)	194 (93.3%)	73 (92.4%)	.797
Height (cm)	167.80 ± 7.79	168.1 ± 7.49	167.0 ± 8.52	.315
Weight (kg)	71.88 ± 11.95	72.26 ± 12.27	70.89 ± 11.08	.386
BMI (kg/m^2^)	25.45 ± 3.45	25.48 ± 3.58	25.35 ± 3.12	.782
Hypertension (*n*, %)	191 (66.6%)	140 (67.3%)	51 (64.6%)	.659
DM (*n*, %)	103 (35.9%)	68 (32.7%)	35 (44.3%)	.067
CVD (*n*, %)	36 (12.5%)	16 (7.7%)	20 (25.3%)	<.001
AF (*n*, %)	19 (6.6%)	8 (3.8%)	11 (13.9%)	.002
PAO (*n*, %)	15 (5.2%)	12 (5.8%)	3 (3.8%)	.503
Smoke (*n*, %)	171 (59.6%)	125 (60.1%)	46 (58.2%)	.773
Drink (*n*, %)	159 (55.4)	117 (56.2%)	42 (53.2%)	.639
SBP (mmHg)	154.56 ± 24.88	153.79 ± 24.08	156.57 ± 26.93	.399
DBP (mmHg)	86.21 ± 14.41	86.13 ± 14.50	86.39 ± 14.27	.893
WBC (×10^9^)	7.42 ± 2.40	6.98 ± 2.01	8.56 ± 2.92	<.001
Neutrophil (×10^9^)	4.43 (3.30, 5.72)	4.25 (3.16, 5.00)	5.38 (3.87, 7.64)	<.001
Lymphocyte (×10^9^)	1.78 ± 0.67	1.86 ± 0.63	1.58 ± 0.72	.002
Monocyte (×10^9^)	0.51 ± 0.17	0.50 ± 0.15	0.53 ± 0.20	.232
FPG (mmol/L)	6.97 ± 2.75	5.61 (5.12, 7.26)	6.30 (5.35, 9.60)	.023
TG (mmol/L)	1.46 (1.08, 2.05)	1.44 (1.07, 2.06)	1.47 (1.08, 2.05)	.869
TC (mmol/L)	4.61 ± 1.36	4.63 ± 1.32	4.55 ± 1.47	.686
HDL‐C (mmol/L)	1.11 ± 0.30	1.10 ± 0.30	1.14 ± 0.32	.279
LDL‐C (mmol/L)	2.99 ± 1.12	3.01 ± 1.08	2.95 ± 1.21	.276
Hcy (μmmol/L)	12.41 (9.88, 16.38)	12.14 (9.83, 14.86)	15.07 (11.22, 20.78)	.001
TOAST (*n*, %)				<.001
LA	176 (61.3%)	107 (51.4%)	69 (87.3%)	
CE	8 (2.8%)	6 (2.9%)	2 (2.5%)	
SA	101 (35.2)	93 (44.7%)	8 (10.1%)	
Others	2 (0.7%)	2 (1.0%)	0 (0%)	
NIHSS	3 (1, 6)	3 (1, 4)	8 (5, 16)	<.001
**SIRI (×10^9^)**	**1.26 (0.79, 1.86)**	**1.18 (0.72, 1.59)**	**1.83 (1.23, 4.41)**	**<.001**

Abbreviations: AF: atrial fibrillation; BMI: body mass index; CE: cardioembolism; CVD: coronary vascular disease; DBP: diastolic blood pressure; DM: diabetes mellitus; FPG: fasting plasma glucose; Hcy: homocysteine; HDL‐C: high‐density lipoprotein cholesterol; LA: large artery atherothrombosis; LDL‐C: low‐density lipoprotein cholesterol; NIHSS: the National Institutes of Health Stroke Scale; PAO: peripheral artery occlusive disease; SA: small‐artery occlusion; SBP: systolic blood pressure; SIRI: systemic inflammation response index; TC: total cholesterol; TG: triglyceride; WBC: white blood cell.

The univariate logistic regression analysis revealed that age (OR = 1.059, *p* < .001), history of CVD (OR = 4.068, *p* < .001) and AF (OR = 4.044, *p* = .004), FPG (OR = 1.106, *p* = .028), Hcy (OR = 1.032, *p* = .005), NIHSS score at admission (OR = 1.523, *p* < .001), AIS subtype, and SIRI (OR = 1.561, *p* < .001) were significantly related to poor function prognosis of AIS (Table 2). These variables were thus introduced into multivariate logistic regression analysis. Although WBC and neutrophil showed a significant correlation with AIS prognosis, WBC and neutrophil were excluded from the multivariate logistic regression model due to the collinearity with SIRI.

**TABLE 2:** Univariate logistic regression analyses of the risk factors significantly associated with poor prognosis

Variables	Odds ratio	95% CI	*p*
Age	1.059	1.033–1.086	<.001
CVD	4.068	1.982–8.351	<.001
AF	4.044	1.562–10.471	.004
FPG	1.106	1.011–1.209	.028
Hcy	1.032	1.009–1.056	.005
WBC	1.306	1.168–1.460	<.001
Neutrophil	1.407	1.244–1.590	<.001
NIHSS	1.523	1.357–1.709	<.001
AIS subtype			<.001
SA	Reference	Reference	
LA	7.496	3.426–16.402	
CE	2.875	0.670–22.426	
**SIRI**	**1.561**	**1.308**–**1.863**	**<.001**

Abbreviations: AF: atrial fibrillation; AIS: acute ischemic stroke; CE: cardioembolism; CVD: coronary vascular disease; FPG: fasting plasma glucose; Hcy: homocysteine; NIHSS: the National Institutes of Health Stroke Scale; SA: small‐artery occlusion; SIRI: systemic inflammation response index; WBC: white blood cell.

Multivariate logistic regression analyses revealed the association between SIRI and poor outcome of AIS (Table 3). In the crude model, the risk of poor prognosis of AIS increased by 2.077 times for each SD increase of SIRI (*p* < .001). After adjusting for age, sex, race, BMI, smoking, drinking, hypertension, diabetes, CVD history, AF history, PAO history, LDL‐C, HDL‐C, FPG, Hcy, NIHSS score at admission, and stroke subtypes, per SD increase of SIRI still caused a 58.9% additional risk of poor outcome (*p* = .037). When dividing SIRI into quartiles, we observed 6.152 times risk for the top quartile against the bottom quartile in the full model, with a significant trend across the quartiles (*p* for trend .006).

**TABLE 3:** Univariate and multivariate logistic regression of SIRI for outcomes

	Odds ratio (95% CI)			
Variables	Univariate	*p*	Multivariate^a^	*p*
SIRI (per SD increase)	2.077 (1.553, 2.777)	<.001	1.589 (1.028, 2.456)	.037
Quartiles of SIRI				
Quartiles 1	Reference		Reference	
Quartiles 2	1.175 (0.465, 2.968)	.734	2.160 (0.592, 7.873)	.243
Quartiles 3	2.594 (1.121, 6.001)	.026	4.919 (1.275, 18.976)	.021
Quartiles 4	6.660 (2.958, 14.995)	<.001	6.152 (1.567, 24.161)	.009
P for trend		<.001		.006

Abbreviations: SD: standard deviation; SIRI: systemic inflammation response index.

^a^ Adjusted for age, sex, race, body mass index, smoking, drinking, hypertension, diabetes, coronary vascular disease history, atrial fibrillation history, peripheral artery occlusive disease history, low‐density lipoprotein cholesterol, high‐density lipoprotein cholesterol, fasting plasma glucose, homocysteine, NIHSS score at admission, and stroke subtypes.

To further explore the ability of SIRI to predict poor prognosis in AIS, we performed ROC and reclassification analyses (Table 4). The area under the curve (AUC) of SIRI for poor outcome of AIS was 0.714 (95% CI: 0.658–0.765, *p* < .001). The sensitivity and specificity values were 68.35% and 71.15%, respectively. The cutoff value of SIRI was 1.349. After adding SIRI into several clinical risk factors (age, sex, race, BMI, hypertension, diabetes, CVD, AF, smoking, drinking, LDL‐C, HDL‐C, and Hcy), AUC increased significantly (0.829 vs. 0.790, *p* for comparison = .016). Moreover, both the category‐free NRI (0.761, 95% CI: 0.517, 1.004, *p* < .001) and IDI (0.093, 95% CI: 0.0512, 0.134, *p* < .001) showed a significant improvement to predict the risk of poor outcome of AIS when adding SIRI into clinical risk factors.

**TABLE 4:** ROC and reclassification analyses for SIRI to evaluate the poor outcome risk of AIS

Model	AUC (95% CI)	*p*	*p* for comparison	NRI	*p*	IDI	*p*
SIRI	0.714 (0.658, 0.765)	<.001	–				
Clinical risk factors^a^	0.790 (0.739, 0.836)	<.001	Reference	Reference	Reference	Reference	Reference
Clinical risk factors + SIRI	0.829 (0.781, 0.871)	<.001	.016	0.761 (0.517, 1.004)	<.001	0.093 (0.0512, 0.134)	<.001

Abbreviation: SIRI: systemic inflammation response index.

^a^ Clinical risk factors: age, sex, race, body mass index, hypertension, diabetes, coronary vascular disease, atrial fibrillation; smoking, drinking, low‐density lipoprotein cholesterol, high‐density lipoprotein cholesterol, and homocysteine.

## 4 DISCUSSION

The present study, for the first time, evaluates the association between SIRI on admission and the 3‐month prognosis of first‐ever AIS. Our results suggest that elevated SIRI is independently associated with poor outcome of AIS. Furthermore, our findings implicate the potential usefulness of SIRI to refine the risk stratification of poor prognosis of AIS. Our work provides clinical evidence for mechanical studies related to the correlation between inflammation and AIS. Most importantly, our present study suggests a potential simple method to optimize the risk stratification and prevent unfavorable functional outcomes of AIS by estimating systemic inflammation status.

SIRI is a newly proposed index to represent systemic inflammation levels by taking three principal inflammatory cells into consideration, namely neutrophil, lymphocyte, and monocyte (Qi et al., 2016). The peripheral circulating neutrophil is one of the earliest inflammatory cells to enter the ischemic parenchyma after AIS, aggravating brain damage by releasing inflammatory mediators (Herz et al., 2015; Jickling et al., 2015). A higher neutrophil level in the early stage of AIS is correlated with a larger infarct volume (Buck et al., 2008). When reducing neutrophils or inhibiting their infiltration into ischemic brain tissue, cerebral infarction volume decreases, showing a protective effect on the brain (Bowes et al., 1995; Chopp et al., 1996; Garau et al., 2005). Monocyte is another important inflammatory cell that aggravates the inflammatory response after AIS. Monocytes in peripheral blood circulation can infiltrate the ischemic tissue after AIS, expanding the brain injury range (Kaito et al., 2013). However, different from neutrophils and monocyte, some lymphocytes mainly play a protective role in the inflammatory response after AIS, regulating and inhibiting the local inflammatory response (Liesz et al., 2013). Therefore, increased neutrophil, monocyte, and reduced lymphocyte may indicate a severe inflammation status and be more harmful to ischemic brain tissues. Accordingly, SIRI, defined as neutrophil count × monocyte count/lymphocyte count, can be a valuable indicator of inflammation status in AIS. Therefore, we hypothesized that SIRI was correlated with the prognosis of AIS.

The results confirmed our hypothesis. In multivariate logistic regression models, elevated SIRI levels were significantly correlated with poor prognosis of AIS, even after adjusting for demographic factors and several clinical risk factors, indicating the independent ability of SIRI for predicting functional prognosis of AIS. Consistently, ROC analyses also showed that SIRI could effectively predict the poor prognosis of AIS. The AUC of SIRI towards the poor outcome of AIS was significant (*p* < .001). After introducing SIRI to several clinical risk factors, the ability to identify the poor prognosis of AIS was significantly improved (AUC: 0.829 vs. 0.790, *p* < .001). However, although ROC analysis is the most commonly used method to evaluate the potential value of new markers, it can be insensitive when discriminating between two models (Cook, 2007; Pencina et al., 2008). Therefore, ROC analysis may be inadequate to estimate the adding value of an indicator. To investigate the value of a new indicator more accurately, statisticians have proposed two new metrics, namely, NRI and IDI, to evaluate the incremental value of a novel biomarker to an existing model (Grunkemeier & Jin, 2015; Pencina et al., 2011; Pickering & Endre, 2012). In this study, both NRI and IDI showed significant improvement in stratifying the risk of poor outcomes after AIS when adding SIRI into the established risk factors model. In conclusion, our result supports the usefulness of SIRI to predict and evaluate the risk of poor prognosis after AIS. The clinical risk factors model combining the SIRI had a more favorable discrimination ability. Therefore, clinicians may improve their medical strategy to prevent poor outcomes after AIS by introducing SIRI into clinical applications.

Several previous studies have demonstrated that SIRI is a valuable index for predicting the prognosis and stratifying the risk of multiple inflammation‐related diseases, which was in accordance with our findings. Qi et al.’s (2016) study prospective randomized controlled trial confirmed SIRI as a useful prognosis predictor in patients with pancreatic adenocarcinomas who received chemotherapy. In Qi et al.’s study (2016), a higher SIRI was independently correlated with elevated interleukin 10 levels, a shorter time to progression, and shorter overall survival after gemcitabine‐based chemotherapy. In Chen et al.’s study (2019), propensity score‐matched analysis exhibited that advanced renal clear cell carcinoma patients with higher SIRI levels showed poorer cancer‐specific survival and overall survival than those with lower SIRI levels. Furthermore, SIRI can also independently predict the prognosis of patients with metastatic pancreatic cancer. The low SIRI level group's overall survival time and disease progression‐free survival time were significantly better than those in the high SIRI level group (Pacheco‐Barcia et al., 2020). Moreover, SIRI can effectively optimize the risk stratification of hyperuricemia (Chen et al., 2021). The study of Chen et al. (2021) included 8095 patients from the Northeast China Rural Cardiovascular Health Study to detect the level of serum uric acid and analyze the relationship between SIRI and hyperuricemia. The results showed a linear relationship between SIRI and the prevalence of hyperuricemia. A higher SIRI level was significantly correlated with an increased prevalence of hyperuricemia (Chen et al., 2021). Recently, Zhang et al. (2020) analyzed 178 aneurysmal subarachnoid hemorrhage patients who underwent surgery. Their result also supported the independent correlation of SIRI with unfavorable outcomes in subarachnoid hemorrhage (Zhang et al., 2020). However, although AIS, tumor, and subarachnoid hemorrhage all have inflammatory mechanisms in common, the correlation between SIRI and AIS has not been reported. For the first time, our study reveals the potential relationship between SIRI and the prognosis of AIS.

In accordance with the present results, previous studies revealed that increased concentration of inflammatory biomarkers, including CRP, IL‐6, and TNF‐α, is associated with increased risks of the occurrence and worse prognosis of stroke (Esenwa & Elkind, 2016). In addition, a study by Tu et al. (2018) reported that decreased concentration of irisin, the level of which was inversely correlated with hs‐CRP and IL‐6, was linked to the poor outcome of AIS. However, these indicators, including CRP, IL‐6, TNF‐α, and irisin, rely on specific laboratory testing programs and are not included in the routine biochemistry test, thus are unconventional to obtain, especially in the primary care condition. Moreover, laboratory testing for CRP, IL‐6, TNF‐α, and irisin is not routinely performed for all patients, especially in primary hospitals. Since SIRI was calculated by neutrophil count × monocyte count/lymphocyte count, the data can be generated from a blood routine examination. Therefore, SIRI is simple and easy to acquire.

Based on our findings and prior studies, anti‐inflammation and immunoregulation therapy might be an available treatment for AIS. Anti‐inflammatory strategies targeting the deleterious processes mediated by inflammatory indicators exhibited a beneficial effect on stroke pathology and improved outcomes (Matsuo et al., 1995; Siniscalchi et al., 2016; Yu et al., 2013). In the experimental stroke model, inhibition of CCR2+, which was highly expressed by a subset of monocytes, showed a protective effect on cerebral ischemia damage by producing anti‐inflammatory cytokines, attenuating the infarct volume and brain edema (Bao et al., 2010; Dimitrijevic et al., 2007; Tsukuda et al., 2011). Antibodies targeting decreasing leukocyte function, reducing neutrophils infiltration, or depleting neutrophils have also shown beneficial effects on stroke pathology by reducing free radical generation and attenuating brain edema after ischemic reperfusion injury (Matsuo et al., 1995; Yu et al., 2013). Therefore, for patients with elevated SIRI at admission, anti‐inflammation and immunomodulatory therapy should be considered.

The present work still has some limitations. First, since our work is a single‐center study with limited sample size and a short‐time follow‐up, large‐scale multicenter studies and long‐term follow‐up are needed to confirm the relationships. Second, all present study participants were Han Chinese, and whether our findings can also be generalized to all patients with first‐ever stroke remains to be determined. Third, although our results illustrated the relationship between the SIRI at admission and the prognosis of AIS, the dynamic change of SIRI over the course of hospitalization was undetermined. In addition, data on potential confounding factors, including dietary intake, and outdoor physical activity, were not obtained. As in any observational epidemiologic study, residual confounding by uncollected risk factors is a source of bias. Larger studies with more confounders for the association between SIRI and AIS are needed in the future. Finally, some defined inflammatory indicators, such as IL‐1, IL‐6, and TNF‐α, were not evaluated in the present study. The correlation between SIRI and these inflammatory indicators, including IL‐1, IL‐6, and TNF‐α, was undetermined.

## 5 CONCLUSION

In summary, for the first time, the present study demonstrates that SIRI is an independent prognostic predictor for the 3‐month functional outcome of AIS. Elevated SIRI was significantly correlated with poor functional prognosis. Our work provides clinical evidence for basic research regarding the association between inflammation and AIS. Most importantly, our work paves an economical and convenient way to refine the risk stratification of poor outcome of AIS, especially in primary healthcare conditions. ]

## CONFLICT OF INTEREST

The authors declare that they have no conflict of interest.

## AUTHOR CONTRIBUTIONS

Yaping Zhou was mainly involved in study design, data acquisition, data analysis, and manuscript preparation. Mingming Cui, Yidi Zhang, and Yuming Zhang were mainly engaged in data acquisition. Xiuli Shang was mainly involved in study design, project administration, funding acquisition, supervision, and validation. All the authors read and approved the final version of the manuscript.

### PEER REVIEW

The peer review history for this article is available at https://publons.com/publon/10.1002/brb3.2619

## Data Availability

The data that support the findings of this study are available from the corresponding author upon reasonable request.
